# Brain oscillations reflecting pain-related behavior in freely moving rats

**DOI:** 10.1097/j.pain.0000000000001069

**Published:** 2017-09-25

**Authors:** Weiwei Peng, Xiaolei Xia, Ming Yi, Gan Huang, Zhiguo Zhang, Giandomenico Iannetti, Li Hu

**Affiliations:** a College of Psychology and Sociology, Shenzhen University, Shenzhen, China; b CAS Key Laboratory of Mental Health, Institute of Psychology, Beijing, China; c Department of Psychology, University of Chinese Academy of Sciences, Beijing, China; d Faculty of Psychology, Southwest University, Chongqing, China; e Neuroscience Research Institute and Key Laboratory for Neuroscience, Ministry of Education/National Health and Family Planning Commission, Peking University, Beijing, China; f School of Biomedical Engineering, Shenzhen University, Shenzhen, China; g Department of Neuroscience, Physiology and Pharmacology, University College London, United Kingdom

**Keywords:** Brain oscillations, Pain, Animal models, Electrocorticography, Gamma-band event-related synchronization (γ-ERS)

## Abstract

Recording oscillatory brain activity holds great promise in pain research. However, experimental results are variable and often difficult to reconcile. Some of these inconsistencies arise from the use of hypothesis-driven analysis approaches that (1) do not assess the consistency of the observed responses within and across individuals, and (2) do not fully exploit information sampled across the entire cortex. Here, we address these issues by recording the electrocorticogram directly from the brain surface of 12 freely moving rats. Using a hypothesis-free approach, we isolated brain oscillations induced by graded nociceptive stimuli and characterized their relation to pain-related behavior. We isolated 4 responses, one phase-locked event-related potential, 2 non–phase-locked event-related synchronizations, and one non–phase-locked event-related desynchronization (ERD), in different frequency bands (δ/θ-ERD, θ/α–event-related synchronization, and gamma-band event-related synchronization). All responses except the δ/θ-ERD correlated with pain-related behavior at within-subject level. Notably, the gamma-band event-related synchronization was the only response that reliably correlated with pain-related behavior between subjects. These results comprehensively characterize the physiological properties of the brain oscillations elicited by nociceptive stimuli in freely moving rodents and provide a foundational work to improve the translation of experimental animal findings to human physiology and pathophysiology.

## Abstract 2

Supplemental Digital Content is Available in the Text.

We comprehensively characterized the physiological properties of pain-related brain oscillations in freely moving rats and provided a foundation for the animal-to-human translation of experimental findings.

## 1. Introduction

Animal models are widely used to explore the pathophysiological mechanisms of chronic pain and to identify novel analgesic compounds.^37,38^ However, only appropriate models permit a successful translation of experimental findings into effective clinical analgesics for humans. Recently, after considering the important differences in sensitivity of the auditory and nociceptive systems between rodents and humans,^19^ we have demonstrated that recording laser-evoked electrocorticographical (ECoG)^4^ responses in freely moving rats is a valid model to assess the function of C-fibre afferent pathways, provided that the activation of the auditory system by the laser thermoacoustic phenomenon is avoided.^62^ This animal model holds considerable promise, as many chronic pain conditions are consequent to a dysfunction of the unmyelinated afferent system.^30,43,55^

Several studies have investigated the relationship between brain responses elicited by nociceptive stimuli and subjective pain reports in humans. Whether neural processes specifically reflecting the emergence of painful percepts can be isolated in the measured brain activity is heavily debated.^16,22,23,41,61^ Indeed, pain-specific processes are difficult to detect, given that (1) the cortical response elicited by transient nociceptive stimuli is dominated by supramodal activities related to the detection of behaviorally relevant salient events,^41^ and (2) nociceptive and non–nociceptive somatosensory neurons are intermixed,^27,63^ and therefore generate signals largely overlapping at the macroscopic scale of EEG or functional magnetic resonance imaging.^28,41,45^ For example, when detected in the time domain, the electrocortical responses elicited by laser stimuli (laser-evoked potentials, LEPs) largely reflect the activity of nonnociceptive–specific neurons,^41^ although they can indirectly provide information about afferent activity in the nociceptive pathways and perceived pain.^16,42^ By contrast, non-phase–locked modulations of ongoing brain oscillations induced by the same nociceptive stimuli have the potential of reflecting more selectively nociceptive processing. For example, gamma-band event-related synchronization (γ-ERS) seems to relate to cortical activities at the interface between stimulus-driven and top–down determinants of pain perception.^12,52,53,66^ However, because the signal-to-noise ratio of non-phase–locked brain oscillations is rather low when they are measured using scalp EEG, their functional properties and selectivity for nociceptive processing are still largely unknown.

In the present study, we provide a comprehensive characterization of brain oscillations induced by nociceptive stimulation, as well as of their significance in relation to behavior, using direct recording from the cortex of 12 awake and freely moving rats. We correlate these brain responses with pain-related behavior both at single-subject level, using a trial-by-trial analysis, and across subjects, using across-trial averages. We discuss the results in relation to the human literature on brain oscillations and pain perception, particularly in the context of translating experimental animal findings to humans.

## 2. Methods

### 2.1. Animal preparation, surgical procedures, and experimental paradigm

The experiments were conducted on 12 adult male Sprague-Dawley rats weighing between 300 and 400 g. Rats were free-choice fed with water and food and were housed in separate cages under temperature- and humidity-controlled conditions. All rats were kept in a 12-hour day–night cycle (light on from 08:00 to 20:00). The local ethics committee approved the surgical and experimental procedures, which adhered to the guidelines for animal experimentation.

During surgery, 14 holes were drilled on the skull at standard stereotaxic locations.^56^ Stainless steel screws (outside diameter = 1 mm) were inserted into the holes without penetrating the dura mater, as ascertained by visual inspection after the animals were sacrificed. Twelve screws were used as active electrodes, and the 2 remaining screws were used as reference and ground electrodes. The detailed positions and coordinates of all electrodes are shown in the supplementary Figure 1 of Ref. 19 (available online at http://links.lww.com/PAIN/A143). After surgery, rats were kept in their cages for at least 7 days before the collection of ECoG data.

During the ECoG data collection, rats were placed into a plastic chamber (length × width × height: 30 cm × 30 cm × 40 cm), within which they could freely move. Radiant-heat nociceptive stimuli generated by an infrared neodymium: yttrium–aluminum–perovskite (Nd:YAP) laser with a wavelength of 1.34 μm (Electronical Engineering, Italy) were delivered on the animal paws through the holes (5-mm diameter) on the floor of the chamber, when the animal was spontaneously still. Nd:YAP laser pulses selectively activate nociceptive terminals located in the most superficial skin layers.^58^ The laser beam was transmitted through an optic fibre, and its diameter was set at ∼4 mm (surface area ∼13 mm^2^) by focusing lenses. Ten laser pulses, each with a duration of 4 milliseconds, were delivered to each of 4 stimulation sites (left forepaw, right forepaw, left hind paw, and right hind paw) using 5 stimulus energies (E1-E5, from 1 J to 4 J, in steps of 0.75 J), for a total of 200 pulses. The order of stimulation sites and stimulus energies was pseudorandomized; the interstimulus interval was never less than 30 seconds, and the target of the laser beam was changed after each stimulus to avoid nociceptor fatigue or sensitization.^31^ Animals were video recorded throughout the experiment, and pain-related behaviors elicited by the laser stimuli were quantified using a 0 to 4 numerical rating scale (NRS) according to previously defined criteria,^8,9^ as follows: 0: no movement; 1: head turning, including shaking or elevating the head; 2: flinching, that is, a small abrupt body jerking movement; 3: withdrawal, involving paw retraction from the nociceptive stimulus; 4: licking the stimulated body territory and whole-body movement. The rating procedure was conducted by an experimenter masked to both stimulus energy and stimulation site. Scores of pain-related behavior were compared using a 2-way (5 × 4) repeated measures analysis of variance (ANOVA), with “stimulus energy” (5 levels: E1-E5) and “stimulation site” (4 levels: left forepaw, right forepaw, left hind paw, and right hind paw) as within-subject factors.

To avoid the activation of the auditory system by the laser-generated ultrasounds,^19^ white noise was played throughout the experiment. This important procedure allows for the selective recording of cortical responses related to the activation of the nociceptive system.^62^

### 2.2. Electrocorticographical data collection and time-domain analysis

Electrocorticographical data were amplified and recorded (Brain Products; high pass: 0.01 Hz; sampling rate: 1000 Hz) and preprocessed using EEGLAB.^6^ Continuous ECoG data were bandpass filtered between 1 and 100 Hz, and notch filtered between 49 and 51 Hz. Epochs were extracted using a window analysis time of 2000 milliseconds (500 ms prestimulus and 1500 ms poststimulus) and baseline corrected using the prestimulus interval. Trials whose amplitude exceeded ± 500 μV in any point of the timecourse and at any electrode were considered to be contaminated by gross artifacts and were manually rejected.^50,57^ An average of 3 ± 6 trials (1.6% ± 3% of the total number of trials) were removed from each subject. For each animal and each stimulation site (left forepaw, right forepaw, left hind paw, and right hind paw), epochs were averaged across trials and time locked to the stimulus onset.

### 2.3. Time–frequency analysis

Time–frequency distributions (TFDs) of ECoG responses were calculated using a windowed Fourier transform with a fixed 200-ms Hanning window.^66^ This yields, for each epoch, a complex time–frequency spectral estimate F(*t*, f) at each time–frequency point, extending from −500 to 1500 ms (in steps of 2 ms) in latency, and from 1 to 100 Hz (in steps of 1 Hz) in frequency. The spectrogram, P(*t*, f)=|F(*t*, f)|^2^, represents the power spectral density as a joint function of time and frequency at each time–frequency point. When the time–frequency analysis is performed on the waveforms averaged across trials in the time domain, the resulting TFDs only contain brain responses phase locked to stimulus onsets.^40^ When performed on single-trial ECoG responses, the resulting TFDs contain brain responses both phase locked and non–phase locked to stimulus onsets.^40^ Spectrograms were baseline corrected by subtracting the average power within the prestimulus reference interval (−400 to −100 ms relative to stimulus onset) for each frequency. This subtraction approach avoids the positive bias introduced when baseline correction is performed using the percentage approach.^20^ The reference interval was chosen to avoid the bias consequent to the windowing of poststimulus activity and padding values.

### 2.4. Isolating brain oscillations

To isolate different features within the single-trial TFDs of all active electrodes, we adopted a data-driven approach based on principal component (PC) analysis (PCA) decomposition with Varimax rotation, which has been proven effective to separate physiologically distinct EEG oscillatory features within the time–frequency domain.^2,3,35^ Such PCA decomposition was performed separately on TFDs at low (1-50 Hz) and high (51-100 Hz) frequencies, as well as on the responses elicited by forepaw and hind paw stimulations.

Time–frequency distributions of single-trial epochs from all electrodes were arranged as vectors and stacked to form a single matrix, which was decomposed into a set of PCs. The obtained PCs were further rotated using the Varimax algorithm, which maximizes the sum of the variances of the squared loadings, thus allowing for an optimal description of the matrix by a linear combination of few basis functions.^25,26^ On the basis of the explained signal variance, 3 PCs were selected for low-frequency TFDs, and 1 PC for high-frequency TFDs. These PCs were rearranged in three-dimensional matrices. The number of PCs was thus determined in a data-driven fashion, as the selected PCs explained the largest amount of variance (>4% for each selected PC, whereas <3% for all remaining PCs). Notably, the 3 main low time–frequency features (low-frequency event-related potential [ERP], event-related desynchronization [ERD], and ERS) and the single main high time–frequency features (high-frequency ERS) identified by PCA were consistent with what is reported in previous human studies.^39,48,54,66^

To identify signal changes significantly different from noise, we conducted the following statistical analysis for each PCA-isolated time–frequency feature. First, we performed a bootstrapping test to identify poststimulus signal changes significantly different from the prestimulus interval, for each time–frequency point. The null hypothesis was that there was no difference between the mean of prestimulus and poststimulus values. The pseudo-*t* statistic between the 2 populations was calculated, and its probability distribution was estimated by permutation testing (5000 times). The distribution of the pseudo-*t* statistics from the baseline population was obtained, and the bootstrap *P* values for the null hypothesis were generated. To account for multiple comparisons, the significance level was corrected using a false discovery rate (FDR) procedure.^1^ Second, the TFD of each selected PC was thresholded using a cutoff at 2 SDs from the mean of all time–frequency points.^21,35^ Third, we extracted the conjunction of time–frequency points whose amplitudes were (1) significantly different relative to the baseline interval and (2) above (for ERP and ERS) or below (for ERD) 2 SDs from the mean of all time–frequency points. Finally, the scalp topography of each isolated time–frequency feature was computed by spline interpolation.

### 2.5. Estimating single-trial parameters of brain oscillations

To estimate automatically the single-trial magnitudes of each isolated time–frequency feature, we used a multiple linear regression with dispersion term (MLR_d_) (^17^ for time-domain responses) that we recently extended for time–frequency brain responses.^21^ This TF-MLR_d_ method takes into account not only the variability in latency and frequency of each TF feature, but also its variability in morphology, that is, the spread along the time and frequency dimensions. This approach effectively enhances the signal-to-noise ratio, thus providing an accurate but still unbiased estimation of each time–frequency feature at a single-trial level.^21^ Estimating such parameters for each single trial allows performing statistical comparisons at the within-subject level. In the context of the current experiment, this allows the exploration of the functional significance of these TF response features in relation to pain-related behavior. Single-trial parameters of each time–frequency feature were obtained from electrodes where the magnitude of the feature was maximal: the central electrodes contralateral to the stimulated territory for ERP, δ/θ-ERD, and γ-ERS (FR2 and PR1 for left forepaw and hind paw stimulations; FL2 and PL1 for right forepaw and hind paw stimulations), and posterior electrodes PL2 and PR2 for θ/α-ERS (for all stimulation sites). (It should be noted that even if the scalp topography of γ-ERS was widespread, its magnitude was maximal over central electrodes contralateral to the stimulated forepaw, and over central midline electrodes when stimulating the hind paw. This topography is in line with previous human findings and suggests that γ-ERS is at least partly generated in the primary sensorimotor cortex contralateral to the stimulated body territory.^12,66^ This was the rationale to assess the relationship between pain-related behaviors and γ-ERS using the pair of central electrodes contralateral to the stimulated side).

### 2.6. Dependency of brain oscillations on stimulus energy

To quantitatively assess the modulation of the oscillations by stimulus energy, we used PCA to isolate the main time–frequency responses for each stimulus energy (E1-E5), by multiplying each PC by its corresponding factor loading (The factor loading of a given PC is a measure of how much that PC contributes to explain the original signal^7^). Significant time–frequency regions were identified by the same conjunction analysis described above (ie, extracting time–frequency points whose amplitudes were significantly different relative to the baseline interval, and also above or below 2 SDs from the mean of all time–frequency points for all stimulus energies^35^). At each energy, when significant time–frequency regions were detected, their scalp topographies were computed by spline interpolation. For each time–frequency feature, we calculated the relative strength of its magnitude at each stimulus energy by dividing the corresponding squared factor loading by the sum of squares of factor loadings across all stimulus energies (E1-E5). The resulting strength values, expressed as percentage, are displayed using radar plot for each feature and stimulation site.

To assess the dependency of time–frequency responses on stimulus energy, their single-subject magnitudes were compared using a 1-way repeated measures ANOVA, with stimulus energy as within-subject factor (5 levels), separately for each stimulation site. When the main effect of the ANOVA was significant, post hoc pairwise comparisons (paired *t* tests with Bonferroni correction) were performed.

### 2.7. Exploring the relationship between brain oscillations and pain-related behavior

To explore the within-subject, trial-by-trial relationship between brain oscillations and pain-related behavior, we estimated the magnitude of each of the TF features isolated with PCA and then correlated them with the corresponding scores of pain-related behavior, using Pearson *r*. The obtained correlation coefficients were transformed to z values using the Fisher *r*-to-z transformation and compared against zero using a one-sample *t* test. To account for multiple comparisons of different stimulation sites, the significance level was adjusted using an FDR procedure for each TF feature.^1^

**Figure 1.**
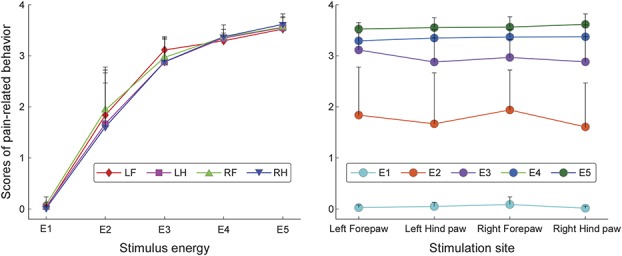
Effect of stimulus energy and stimulation site on pain-related behavior. Radiant-heat laser stimuli of 5 energies (E1-E5) were delivered to 4 anatomical sites (LF, left forepaw; LH, left hind paw; RF, right forepaw; RH, right hind paw). Error bars represent the SEM across subjects. Note that scores of pain-related behavior were significantly modulated by stimulus energy, but not by stimulation site.

We also explored the across-subject relationship between the brain oscillations and pain-related behavior. This analysis assessed whether the variability of pain-related behaviors across different animals is reflected in the time–frequency features of the brain response elicited by the laser stimuli. Such between-subject analysis requires a certain amount of variability in the average pain-related behavior across subjects. When carefully observing the across-subject variability of pain-related behavior elicited by laser stimulation at different stimulus energies (from E1 to E5), it was clear that the variability of the behavioral responses was maximal when elicited by E2, and moderate when elicited by E3, whereas the other energies showed a floor (E1) or a ceiling (E4 and E5) effect (supplementary Fig. 1, available online at http://links.lww.com/PAIN/A486). Therefore, we first used the ECoG responses elicited by nociceptive stimuli delivered using E2 (2.25 J), that is, the energy at which the between-subject variability of pain-related behavior was the highest (Fig. 1). Average magnitudes of different time–frequency features were calculated for each animal and stimulated site. The correlations between estimated parameters and behavioral scores were expressed as Pearson *r* values. To account for multiple comparisons of different stimulation sites, the significance level was FDR corrected for each TF feature.^1^ We also verified the reliability of the obtained results by performing the same across-subject correlation analysis using the data elicited by nociceptive stimuli delivered at energies E2 and E3.

The relationship between stimulus energy and pain-related behavior was not linear (Fig. 1 and supplementary Fig. 1, available online at http://links.lww.com/PAIN/A486). Importantly, the relationship between stimulus energy and the magnitude of brain oscillations was not linear either. For example, the increase of pain-related behavior and γ-ERS magnitude from E4 to E5 was significantly smaller than that from E2 to E3 (Fig. 1). The similarity of these nonlinear relationships was the rationale to assess the relationship between pain-related behavior and the magnitude of brain oscillations using Pearson *r* linear correlation analysis.

Given that such nonlinear relationship between stimulus energy and pain-related behavior may nevertheless affect the association between pain-related behavior and brain oscillations, we also modeled the nonlinear relationship between stimulus energy and pain-related behavior using a quadratic polynomial function (supplementary Fig. 2, available online at http://links.lww.com/PAIN/A486). We adjusted the scores of pain-related behavior using the modeled quadratic polynomial function and assessed their relationship with the magnitudes of each time–frequency feature using the linear correlation analysis described above (in the same section), at both within-subject and between-subject levels.

## 3. Results

### 3.1. Pain-related behavior

As expected, scores of pain-related behavior were strongly determined by the energy of the nociceptive stimulation (F = 161.2, *P* < 0.001, partial eta-squared 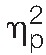 = 0.93, Fig. 1), with more intense behavior at high-stimulus energies. By contrast, the intensity of pain-related behavior was not different at the 4 stimulation sites (F = 1.94, *P* = 0.14, 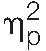 = 0.13; Fig. 1), and there was no significant interaction between stimulus energy and stimulation site (F = 1.52, *P* = 0.19, 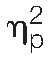 = 0.10).

### 3.2. Laser-induced brain oscillations

**Figure 2.**
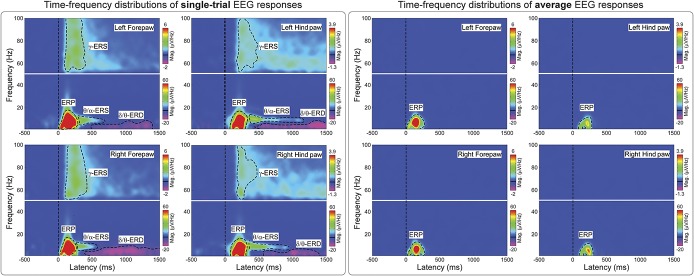
Time–frequency distributions (TFDs) of single-trial and average EEG responses elicited by nociceptive laser stimulation. Left panel*:* Group-level TFDs of single-trial responses. Displayed signals were measured from the 4 central electrodes (FL2, FR2, PL1, and PR1). The color scale represents the increase or decrease of oscillation magnitude, relative to a prestimulus interval (−400 to −100 ms). Time–frequency distributions of single trials contain both phase-locked (ERP: 0-300 ms, 1-20 Hz) and non–phase-locked brain responses (δ/θ-ERD: 500-1500 ms, 1-8 Hz; θ/α-ERS: 300-600 ms, 4-12 Hz; γ-ERS: 100-400 ms, and 50-100 Hz), highlighted by the black dashed lines. Right panel*:* Group-level TFDs of average responses. Displayed signals were measured from the 4 central electrodes (FL2, FR2, PL1, and PR1). Time–frequency distributions of the average responses contain only phase-locked brain responses (ERP), indicating that δ/θ-ERD, θ/α-ERS, and γ-ERS are non–phase-locked to laser stimuli, and cannot be detected after across-trial averaging in the time domain. ERD, event-related desynchronization; ERP, event-related potential; γ-ERS, gamma-band event-related synchronization.

Figure 2 shows the group-level average TFDs of single-trial (left panel) and average (right panel) brain activity elicited by nociceptive stimulation. There were 4 main responses: a strong phase-locked response corresponding to multiple LEP deflections (eg, N2 and P2 waves) visible in the time domain (ERP: 50-300 ms, 1-20 Hz; see also supplementary Figure 3 (available online at http://links.lww.com/PAIN/A486] and^62^), and 3 non–phase-locked responses: a desynchronization of delta or theta band oscillations (δ/θ-ERD: 500-1500 ms, 1-8 Hz), a synchronization of theta or alpha band oscillations (θ/α-ERS: 300-600 ms, 4-12 Hz), and a synchronization of gamma-band oscillations (γ-ERS: 100-400 ms, 50-100 Hz). Only the ERP response was still visible when TFDs were calculated using the brain activity averaged across trials in the time domain (Fig. 2, right panel). This result confirms that the ERP was the only response phase locked to the stimulus onset and that the δ/θ-ERD, θ/α-ERS, and γ-ERS responses were non–phase locked, and therefore only detectable when TFDs are computed on single trials in the time domain.

### 3.3. Data-driven identification of time–frequency responses

**Figure 3.**
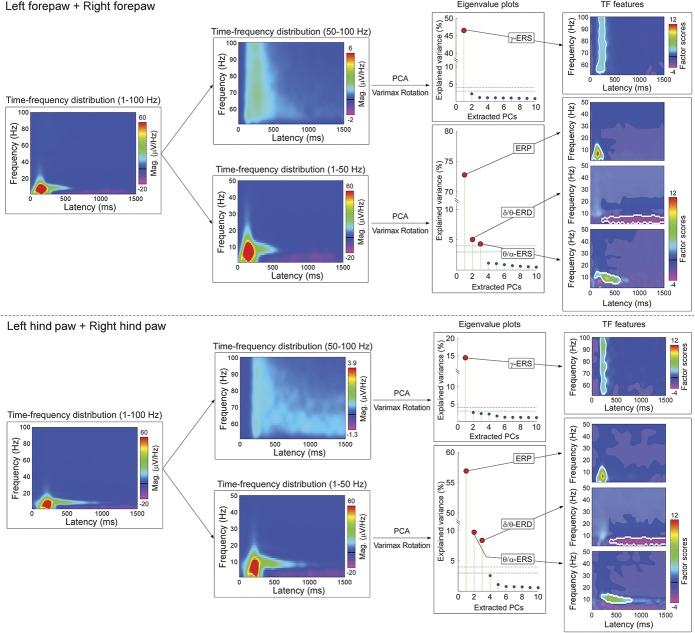
Hypothesis-free approach to isolate time–frequency EEG responses. Time–frequency distributions of single-trial responses measured from all active electrodes (first column) were divided into low frequency (1-50 Hz) and high frequency (51-100 Hz) (second columns), separately for forepaw and hind paw stimulations (top and bottom panels, respectively). Principal component analysis decomposition with Varimax rotation was applied to isolate independent response features. The eigenvalue plots (third column) show the variance explained by the first 10 PCs. For low frequencies, the first 3 PCs corresponded to the ERP (located at 23-275 ms and 1-15 Hz for forepaw stimulation, at 89-305 ms and 1-15 Hz for hind paw stimulation), the δ/θ-ERD (220-1500 ms and 1-8 Hz for forepaw stimulation, 363-1500 ms and 1-8 Hz for hind paw stimulation), and the θ/α-ERS (81-609 ms and 4-14 Hz for forepaw stimulation, 191-807 ms and 6-14 Hz for hind paw stimulation). They explained the largest amount of variance of single-trial TFDs (forepaw stimulation: 72.8%, 5.0%, and 4.3% respectively; hind paw stimulation: 56.9%, 8.4%, and 9.8% respectively). For high frequencies, the first PC corresponded to γ-ERS (119-313 ms and 53-100 Hz for forepaw stimulation and 173-303 ms and 53-100 Hz for hind paw stimulation) and explained the largest amount of variance of single-trial TFDs (forepaw stimulation: 46.6%; hind paw stimulation: 14.4%). The variance explained by any remaining PC was <3%. These PCs were considered as noise and excluded from the following analyses. For each PCA-isolated time–frequency feature (fourth column), a bootstrapping test was performed to isolate poststimulus time–frequency points whose amplitudes were significantly different from the prestimulus interval (not shaded in gray). In addition, TFDs of the PCs were thresholded using a two-SD cutoff (white outlined). Scalp topographies of each PCA-isolated time–frequency features are displayed in the fifth column. γ-ERS, gamma-band event-related synchronization; ERD, event-related desynchronization; ERP, event-related potential; PCA, principal component analysis; TFD, time–frequency distribution.

The time–frequency ECoG responses elicited by the nociceptive stimulus were identified using a hypothesis-free procedure (PCA decomposition with Varimax rotation), illustrated in Figure 3. At low frequencies, 3 PCs contributed to the signal variance above the 4% threshold. These PCs corresponded to the ERP, δ/θ-ERD, and θ/α-ERS (forepaw stimulation: 72.8% [ERP], 5.0% [δ/θ-ERD], and 4.3% [θ/α-ERS]; hind paw stimulation: 56.9% [ERP], 8.4% [δ/θ-ERD], and 9.8% [θ/α-ERS]). At high frequencies, only 1 PC contributed to the variance above the 4% threshold and corresponded to the γ-ERS. The variance explained by any of the remaining PCs was <3%, both at low and high frequencies.

The right part of Figure 3 shows the scalp topographies of the PCA-isolated time–frequency responses. The topography of the ERP response was remarkably similar to that of the N2 wave in the time domain (supplementary Fig. 3, available online at http://links.lww.com/PAIN/A486), with a maximum at central electrodes and a distribution slightly but clearly contralateral to the stimulated territory (Fig. 3). This contralateral distribution was particularly clear following forepaw stimulation, given the larger distance between the cortical representations of the right and left forepaws in the primary sensorimotor cortex.^62^ θ/α-ERS topographies were maximal over posterior regions, whereas δ/θ-ERD and γ-ERS topographies were maximal at central electrodes, again with a distribution slightly contralateral to the stimulated territory.

Given that laser stimuli at energy E1 did not evoke overt pain-related behaviors and clear brain responses, we performed the same PCA with varimax rotation excluding EEG trials at stimulus energy E1. The PCA-decomposed TF features were almost identical to those identified when all stimulus energies were included in the analysis (supplementary Fig. 4, available online at http://links.lww.com/PAIN/A486).

### 3.4. Dependency of brain oscillations on stimulus energy

**Figure 4.**
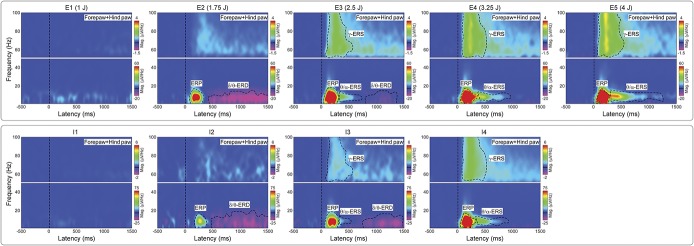
Time–frequency distributions of single-trial EEG responses at different stimulus energies (E1-E5) and different levels of pain-related behaviors (I1-I4). Group-level TFDs of single-trial rat-LEP responses measured from the 4 central electrodes (FL2, FR2, PL1, and PR1) for each stimulus energy (E1-E5, top panel) and each level of pain-related behavior (I1-I4, bottom panel). Responses from the 4 stimulation sites were pooled. The color scale represents the increase or decrease of oscillation magnitude, relative to a prestimulus interval (−400 to −100 ms). Time–frequency features are marked with dashed black lines. Note how the magnitudes of ERP, θ/α-ERS, and γ-ERS increase with both stimulus energy and pain-related behavior. By contrast, the magnitude of δ/θ-ERD is maximal at stimulus E2 and pain-related behavior I2. Pain-related behavior l1: 0≤NRS<1; pain-related behavior l2: 1≤NRS<2; pain-related behavior l3: 2≤NRS<3; pain-related behavior l4: 3≤NRS≤4; ERS, event-related synchronization; ERD, event-related desynchronization; ERP, event-related potential; LEP, laser-evoked potential; NRS, numerical rating scale (0-4); TFD, time–frequency distribution.

Stimulus energy (E1-E5) had important effects on the features of laser-induced brain responses in the time–frequency domain, as shown qualitatively in the top panel of Figure 4. First, response magnitudes were clearly dependent on stimulus energy; although the ERP, θ/α-ERS, and γ-ERS magnitudes increased monotonically with stimulus energies, the δ/θ-ERD magnitude was maximal at the stimulus energy E2. Second, stimulus energy remarkably altered the time–frequency boundaries of the different responses, sometimes in a nonmonotonic fashion: for example, the θ/α-ERS increased in duration with stimulus energy, with its onset–offset latencies changing from 310 to 726 ms to 280 to 1250 ms, and γ-ERS increased from 90 to 483 ms to 81 to 630 ms. It is possible that some of these changes in time–frequency boundaries resulted from the increase of response magnitude with energy of the eliciting stimulus.

When grouping EEG trials according to the ratings of pain-related behavior (I1: 0 ≤ NRS < 1; I2: 1 ≤ NRS < 2; I3: 2 ≤ NRS < 3; I4: 3 ≤ NRS ≤ 4) instead of stimulus energy, the magnitudes of ERP, θ/α-ERS, and γ-ERS monotonically increased with pain-related behavior, whereas the magnitude of δ/θ-ERD was maximal at I2 and decreased at I3 and I4 (bottom panel of Fig. 4). These findings were similar to what we observed when grouping EEG trials according to stimulus energy.

**Figure 5.**
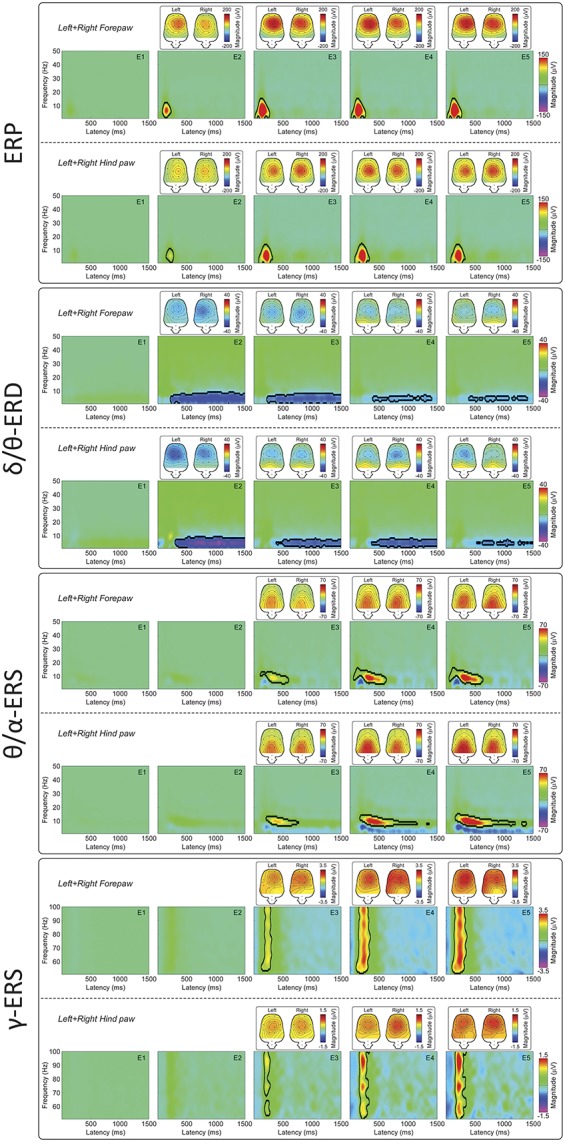
Principal component analysis–isolated time–frequency features at different stimulus energies (E1-E5). Time–frequency distributions of ERP, δ/θ-ERD, θ/α-ERS, and γ-ERS are displayed in the 4 panels. Magnitudes of ERP, θ/α-ERS, and γ-ERS increased with stimulus energy, whereas δ/θ-ERD magnitude was maximal at E2. When significant time–frequency regions were detected, their scalp topographies were displayed. Event-related potential, δ/θ-ERD, and γ-ERS topographies were maximal at central electrodes, with a distribution slightly contralateral to the stimulation site. By contrast, the scalp topography of θ/α-ERS was maximally over the posterior regions, regardless of stimulus energy. ERS, event-related synchronization; ERD, event-related desynchronization; ERP, event-related potential.

Figure 5 quantifies the modulatory effects of stimulus energy on the presence, time–frequency boundaries, and scalp topography of the responses. At high-stimulus energies, all time–frequency responses were statistically significant. Stimulus energy also affected the presence and the time–frequency limits of the responses. This was particularly striking when considering the θ/α-ERS and δ/θ-ERD responses: At E2 there was no θ/α-ERS, whereas the δ/θ-ERD was maximal, lasting almost 1 second (from 500 to 1500 ms poststimulus); at E3 the δ/θ-ERD dramatically decreased, and only remained in the later part of the signal (from 800 to 1400 ms), whereas the θ/α-ERS appeared in the 300 to 600 ms time window. At E4 and E5, the θ/α-ERS increased in both magnitude and duration, and almost took over the time window where the δ/θ-ERD was detected at E2 and E3. Similarly to the θ/α-ERS, the γ-ERS monotonically increased in magnitude and duration from E2 to E5. The ERP was the only response that showed minimal changes in time–frequency size with stimulus energy (Figs. 4 and 5).

Scalp topographies of ERP, δ/θ-ERD, and γ-ERS within the detected significant time–frequency regions were maximal at central electrodes with a distribution slightly contralateral to the stimulation site. By contrast, scalp topographies of θ/α-ERS were maximally distributed at the posterior region, regardless of stimulated side.

**Figure 6.**
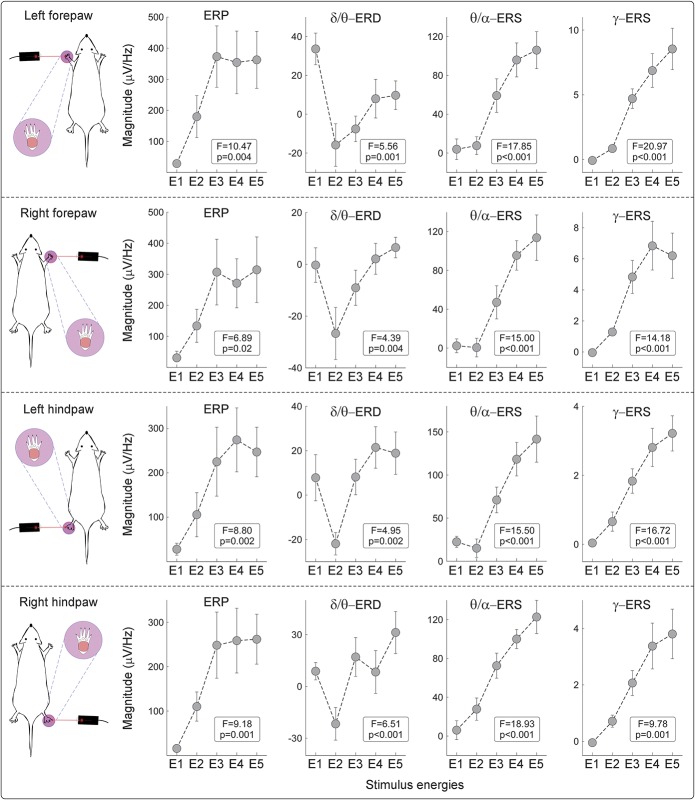
Dependency of single-trial TFD magnitudes on stimulus energy at the within-subject level. Single-trial magnitudes of the isolated TF features were estimated by applying TF-MLR_d_ on TFDs of single-trial rat-LEP responses.^21^ Although ERP, θ/α-ERS, and γ-ERS magnitudes significantly increased with stimulus energy, δ/θ-ERD magnitudes were maximal at stimulus energy E2. Mean values are displayed as gray dots, and error bars represent SEM across subjects. Event-related potential, δ/θ-ERD, and γ-ERS magnitudes were measured at central electrodes contralateral to the stimulated territory (FR2 and PR1 for left forepaw and hind paw stimulations and FL2 and PL1 for right forepaw and hind paw stimulations, respectively), and θ/α-ERS magnitudes were measured at posterior electrodes (PL2 and PR2 for all stimulation sites). ERS, event-related synchronization; ERD, event-related desynchronization; ERP, event-related potential; LEP, laser-evoked potential; TFD, time–frequency distribution.

One-way repeated measures ANOVA revealed that the magnitude of all TF features was significantly dependent on stimulus energy, regardless of stimulation site (Fig. 6 and Table 1). The magnitude modulations of θ/α-ERS and γ-ERS were virtually identical, with a monotonic increase from E2 to E5. The magnitude of the phase-locked ERP plateaued at E3 and remained constant at higher energies, an observation strongly reminiscent of the energy-dependent modulation of the amplitude of the N2 wave observed in the time domain.^62^ The dependence of δ/θ-ERD magnitude on stimulus energy stood out as clearly distinct: δ/θ-ERD was largest at E2, decreased at E3, and almost disappeared at E4-E5 (Fig. 6, supplementary Fig. 5; available online at http://links.lww.com/PAIN/A486, and Table 1).

**Table 1:**
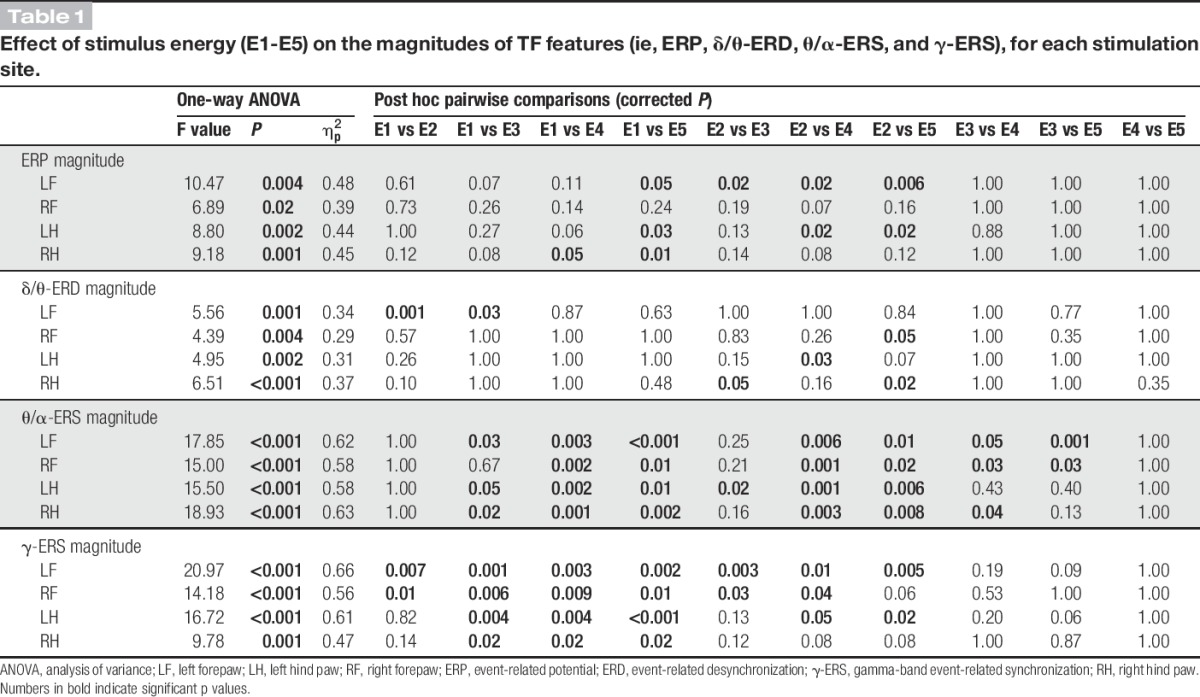
Effect of stimulus energy (E1-E5) on the magnitudes of TF features (ie, ERP, δ/θ-ERD, θ/α-ERS, and γ-ERS), for each stimulation site.

### 3.5. Within-subject, trial-by-trial relationship between brain oscillations and pain-related behavior

Single-trial correlations between the magnitude of each time–frequency response and the corresponding pain-related behavior are summarized in Table 2, for each stimulation site. ERP, θ/α-ERS, and γ-ERS magnitudes were always significantly and positively correlated with pain-related behavior (*r* > 0.33 ± 0.26 and *P* < 0.01 for all correlations). By contrast, no significant correlation was observed between δ/θ-ERD magnitudes and pain-related behavior.

**Table 2:**
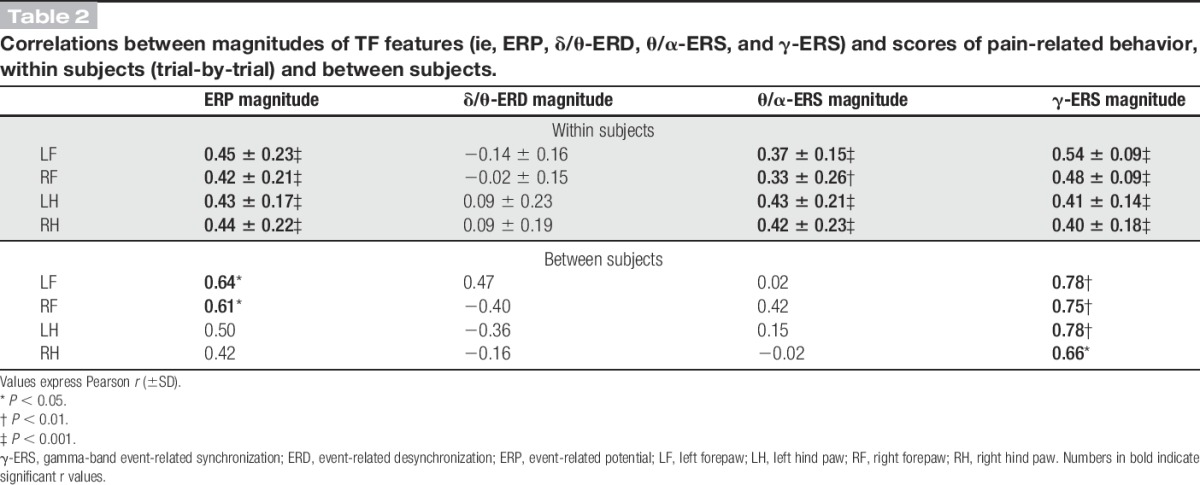
Correlations between magnitudes of TF features (ie, ERP, δ/θ-ERD, θ/α-ERS, and γ-ERS) and scores of pain-related behavior, within subjects (trial-by-trial) and between subjects.

### 3.6. Between-subject relationship between brain oscillations and pain-related behavior

**Figure 7.**
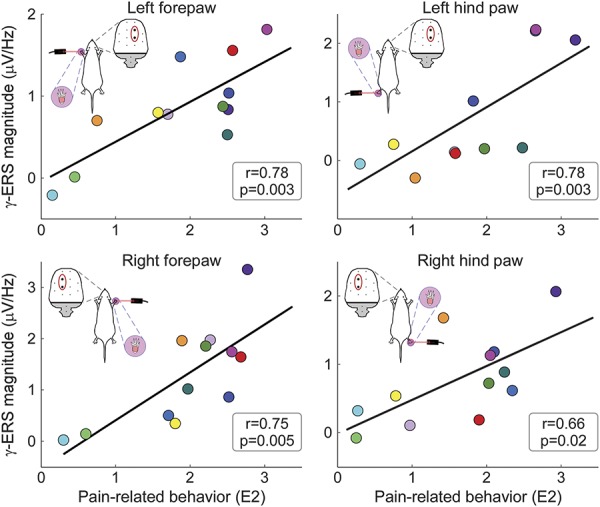
Between-subject correlations between γ-ERS magnitude and pain-related behavior. At stimulus energy E2 (ie, when the variability in pain-related behavior was maximal, Fig. 1), there was a significant relationship between γ-ERS magnitudes and pain-related behavior, for all stimulation sites. Coloured dots represent values of different subjects, and black lines represent the best linear fit. γ-ERS magnitudes were measured at central electrodes contralateral to the stimulated territory (FR2 and PR1 for left forepaw and hind paw stimulations and FL2 and PL1 for right forepaw and hind paw stimulations, respectively). The same results were obtained when also considering trials at energy E3 (see supplementary Fig. 6, available online at http://links.lww.com/PAIN/A486). γ-ERS, gamma-band event-related synchronization.

None of the time–frequency responses reflected pain-related behavior across subjects, with the notable exception of the γ-ERS (left forepaw: *r* = 0.78, *P* = 0.003; right forepaw: *r* = 0.75, *P* = 0.005; left hind paw: *r* = 0.78, *P* = 0.003; right hind paw: *r* = 0.66, *P* = 0.02; Fig. 7 and Table 2). The ERP magnitude correlated with pain behavior only in response to forepaw stimulation (left forepaw: *r* = 0.64, *P* = 0.02; right forepaw: *r* = 0.61, *P* = 0.02, Table 2), but not in response to hind paw stimulation (left hind paw: *r* = 0.50, *P* = 0.10; right hind paw: *r* = 0.42, *P* = 0.17, Table 2). θ/α-ERS and δ/θ-ERD magnitudes were not correlated with pain behavior, for all stimulation sites (−0.36 < *r* < 0.42, *P* > 0.05 for all correlations, Table 2).

When the same across-subject correlations were additionally calculated using data at stimulus energies E2 and E3 (see Methods section), γ-ERS was again the only brain response strongly correlated with pain-related behavior across subjects, at all stimulation sites (supplementary Fig. 6, available online at http://links.lww.com/PAIN/A486). This indicates the robustness of the result observed when only considering trials collected at stimulus energy E2.

These results were also confirmed when scores of pain-related behavior were adjusted using the modeled quadratic polynomial function (supplementary Table 1, supplementary Fig. 7, available online at http://links.lww.com/PAIN/A486). Almost identical to what was observed with the linear correlation analysis, all brain responses, with the notable exception of the δ/θ-ERD, significantly reflected with pain-related behavior at within-subject level, whereas the γ-ERS was the only response that reliably correlated with pain-related behavior also at between-subject level.

## 4. Discussion

Exploring brain oscillatory activity holds great promise in pain research. However, experimental results are variable and often difficult to reconcile. Some inconsistencies arise from the use of analysis approaches that (1) are not data driven, (2) do not assess statistically the robustness of the measured responses within individuals and across individuals, and (3) do not exploit the information provided by all EEG or MEG sensors.^36^ To address these issues, we used a hypothesis-free, data-driven statistical approach based on PCA with Varimax rotation to analyze multichannel ECoG recordings of brain oscillations induced by nociceptive laser stimuli in awake freely moving rats and to characterize their relation to pain-related behavior.

We obtained 4 main findings. First, we isolated 4 distinct oscillatory responses (Figs. 2 and 3): a phase-locked response (ERP), and 3 non–phase-locked responses (δ/θ-ERD, θ/α-ERS, and γ-ERS). Second, both the occurrence and time–frequency boundaries of the non–phase-locked responses changed markedly, and often in a nonmonotonic fashion, with the energy of the applied stimulus. This is the most general conclusion of this study: when relating brain activity with parametric measures of behavior, it is incorrect to estimate the magnitude of stimulus-induced brain oscillations using fixed time–frequency windows, as if these modulations are unitary phenomena. Instead, the use of a blind-source separation algorithm is imperative to isolate and quantify physiologically independent responses that originate in different cortical systems and often overlap in time and space. Third, all responses, with the notable exception of the δ/θ-ERD, correlated with pain-related behavior within subjects. Fourth, the γ-ERS was the only response that reliably correlated with pain-related behavior also between subjects. This comprehensive description of brain oscillatory activity in relation to pain behavior provides a basis for a more effective translation of animal experiments into human pain research, although it is important to remember the different physiological properties of somatosensory systems across species: Laser-evoked responses reflect the activation of C-fibres in rats,^62^ but of both Aδ- and C-fibres in humans.^15^

### 4.1. Phase-locked event-related potential

The phase-locked ERP response at 1 to 20 Hz (forepaw: 23-275 ms; hind paw: 89-305 ms) is the time–frequency counterpart of the LEP deflections observed in the time domain.^21,40^ Its topographical distribution was maximal over the central regions contralateral to the stimulated territory, particularly following forepaw stimulation (Figs. 3 and 5). This topography, together with the strong similarity of the neural generators of human and murine LEPs,^62^ suggests that the phase-locked ERP reflects mixed neural activities from the cingulate cortex, bilateral operculo-insular areas, and primary sensorimotor cortex.^10,24,62^ Single-trial ERP magnitudes were positively related to both stimulus energy (Figs. 4–6 and Table 1) and pain-related behavior, but only within subject (Table 2). In other words, the magnitude of the phase-locked ERP was a good predictor of the intensity of pain-related behavior within subject, but regardless of its absolute amplitude. Thus, it was possible that individuals with overall small ERPs had high scores of pain-related behavior overall, and vice versa. These observations indicate that the phase-locked ERP is functionally similar to the biphasic vertex potential elicited by fast-rising salient stimuli, regardless of their sensory modality,^41^ and therefore it largely reflects supramodal neural processes consequent to the detection of behaviorally relevant changes in the environment.^22,23,41^ Thus, the within-subject correlation with pain-related behavior is likely driven by the saliency content of the stimulus rather than by pain-specific neural activity. Experimental paradigms that dissociate afferent input from stimulus saliency content^22,51,66^ can be used to formally test this hypothesis.

### 4.2. Non–phase-locked δ/θ–event-related desynchronization response

The physiological properties of the non–phase-locked δ/θ-ERD response at 1 to 8 Hz (forepaw: 220-1500 ms; hind paw: 363-1500 ms) were reminiscent of the α-ERD induced by laser stimuli in humans. First, the rat δ/θ-ERD was maximal over the contralateral hemisphere following forepaw stimulation, but centrally distributed following hind paw stimulation (Figs. 3 and 5). This observation suggests that δ/θ-ERD reflects the activation of the primary sensorimotor cortex contralateral to the stimulated territory.^18,44,47^ Second, δ/θ-ERD magnitude was maximal at stimulus energy E2 (Figs. 4–6 and Table 1), and progressively decreased at stimulus energy E2-E5 (Fig. 6), in line with a previous report that the human α-ERD elicited by laser stimulation of C-fibres was maximal at moderate stimulus intensity.^39^ Interestingly, single-trial δ/θ-ERD magnitudes did not correlate with pain-related behavior, either within subjects or between subjects (Table 2). This indicates that the long-lasting δ/θ-ERD response of rat C-LEPs is unlikely to reflect nociceptive-specific or saliency-related neural activity, a hypothesis put forward also for the α-ERD elicited by nociceptive laser stimuli in humans.^18,39^

Altogether, these results show that stimulus-induced reductions in oscillation amplitude occur at lower frequencies in rats (δ/θ) than in humans (α), probably because of inherent species differences. Indeed, EEG frequencies depend, at least partly, on brain size,^29^ and in dogs visual stimuli induce ERD at 2-6 Hz, functionally analogous to the suppressions of oscillations at 10 to 20 Hz in primates.^64^ This difference, probably consequent to the more primitive cytoarchitecture of murine neocortex,^64^ needs to be carefully considered when translating animal experiments to humans.

### 4.3. Non–phase-locked θ/α–event-related synchronization

The non–phase-locked θ/α-ERS at 4 to 14 Hz (forepaw: 81-609 ms; hind paw: 191-807 ms) was always maximal over the occipital region (Figs. 4 and 6). This is strikingly different from human studies,^21,39^ which consistently show that laser-induced ERS occurs at higher frequencies (∼10-20 Hz), and has a frontal scalp distribution. The θ/α-ERS magnitude monotonically increased with stimulus energy (Figs. 4–6 and Table 1), and, importantly, its duration dramatically increased with stimulus energy (from 416 ms at E2 to 970 ms at E5; Fig. 6), which, to the best of our knowledge, has never been reported in human studies.^21,39,48,53^ Similarly to the positive correlation between β-ERS magnitude and pain intensity observed in humans,^16,19^ single-trial θ/α-ERS magnitudes measured in the current study significantly correlated with pain-related behavior within subjects, but not between subjects (Table 2). It is difficult to understand the functional significance of such novel θ/α-ERS response on the basis of the current evidence. Exploring its magnitude modulation and time–frequency–space extension using a number of experimental manipulations, including the previously discussed dissociation of stimulus saliency from afferent input,^22,51,66^ is necessary to clarify this issue.

### 4.4. Non–phase-locked gamma-band event-related synchronization

The non–phase-locked γ-ERS response at 53 to 100 Hz was maximal contralaterally to the stimulated forepaw (119-313 ms), but more centrally distributed when stimulating the hind paw (173-303 ms) (Figs. 3 and 5). This scalp distribution not only rules out the possibility that γ-ERS reflects muscle activity,^11,65^ but also indicates that γ-ERS is, at least partly, generated from the primary sensorimotor cortex contralateral to stimulated side—a finding well documented also in human studies.^12,66^ The trial-by-trial variability of γ-ERS magnitude significantly correlated with pain-related behavior within subjects, for all stimulation sites (Table 2). Although not explicitly tested in the present study, the trial-by-trial correlation of γ-ERS and pain-related behavior is unlikely to reflect stimulus saliency, and it would still be present during experimental manipulations that disrupt the relationship between pain intensity and the magnitude of all other features of the laser-induced EEG responses in humans.^12,66^

The most striking result is that γ-ERS was the only response feature predictive of pain-related behavior *across subjects* (Fig. 7 and Table 2). In other words, measuring a large γ-ERS in a given animal allowed predicting intense pain-related behavior and vice versa. This result was consistent with what we recently observed in human subjects: The largest part of the laser-evoked EEG responses reflects pain reports within subjects, but fails to reflect the variability in pain sensitivity between subjects; whereas γ-ERS reflects pain perception, both within individuals and across individuals (L. Hu and G.D. Iannetti, June 2016, Unpublished data). This suggests that the relationship between the neural activity indexed by γ-ERS and pain variability is phylogenetically conserved across mammals.

Interestingly, γ-oscillations are so far the only brain signal able to track the intensity of tonic painful percepts, both in healthy volunteers^32,46,52^ and patients with chronic pain^33,49^—a result further confirming that the γ-ERS recorded in the current study unlikely reflects stimulus saliency. It is important to highlight that direct causal evidence linking γ-ERS with pain perception is still missing: Although transcranial direct current stimulation modulating γ-oscillations^14,34^ significantly affects pain perception in humans,^13,60^ one must remember that transcranial direct current stimulation affects a large range of brain oscillatory activities, making it difficult to exclude alternative mechanisms than γ-oscillations as the underlying cause. In future studies, the selective modulation of parvalbumin-positive interneurons critical for generating oscillations in the γ-band will be key to precisely addressing this question.^5,59^

## Conflict of interest statement

The authors have no conflict of interest to declare.

This work was supported by the National Natural Science Foundation of China (31500921 to W.W. Peng, 31471082 and 31671141 to L. Hu), the Chongqing Research Program of Basic Research and Frontier Technology (cstc2015jcyjBX0050 to L. Hu), the Scientific Foundation project of Institute of Psychology, Chinese Academy of Sciences (Y6CX021008 to L. Hu), the Wellcome Trust (PAIN JLARAXR to G. Iannetti), and a Consolidator Grant of the European Research Council (PAINSTRAT to G.D. Iannetti). G.D. Iannetti is additionally supported by a fellowship at the Paris Institute for Advanced Studies (France).

## Supplementary Material

SUPPLEMENTARY MATERIAL

## Appendix A. Supplemental digital content

Supplemental digital content associated with this article can be found online at http://links.lww.com/PAIN/A486.
