# Supplementary material for: Brain oscillations reflecting pain-related behavior in freely moving rats
Source: Pain. 2017 Sep 25;159(1):106–18. doi: 10.1097/j.pain.0000000000001069 (PMC5737457; doi:10.1097/j.pain.0000000000001069)
Supplement: SUPPLEMENTARY MATERIAL [file jop-159-106-s002.pdf]

Supplementary Material for

"Brain oscillations reflecting pain-related behavior in freely-moving rats"

EEG analysis in the time domain

Peak latency and amplitude of the main deflection visible in the electrocorticogram (ECoG) in the time-domain (N2 wave) were measured from the average waveform across the four central electrodes (FL2, FR2, PL1, and PR1), as described elsewhere [4]. The scalp topographies were computed by spline interpolation, and the scalp boundaries were determined according to the stereotaxic atlas [1].

Supplementary Fig. 3 shows the group-level laser-evoked responses in the time domain (mean ± SEM), together with the scalp topography of the main negative wave (N2: peaking at 146, 149, 225 and 225 ms for left forepaw, right forepaw, left hindpaw, and right hindpaw, respectively). As previously described, the difference in N2-wave latencies between forepaw and hindpaw stimulation is compatible with the conduction velocity of C afferent fibres [2; 3]. In contrast, the N2-wave scalp topographies were similar across the four stimulated territories, with a maximum around central electrodes, slightly but clearly contralateral to the stimulated territory (Supplementary Fig. 3).

Relative magnitude of laser-induced brain oscillations

The relative strength of the magnitude of the four time-frequency responses at the five stimulus energies is displayed in Supplementary Fig. 5 as radar plots. The responses were isolated using a

data-driven approach based on PCA with Varimax rotation, and their magnitude was subsequently estimated for each trial. Magnitude modulations of the non-phase-locked θ/α-ERS and γ-ERS were virtually identical, with a monotonic increase from E2 to E5. The magnitude of the phase-locked ERP plateaued at E3, and remained constant at higher energies. The δ/θ-ERD stood out as clearly distinct: its magnitude was largest at E2, importantly decreased at E3, and became barely detectable at E4-E5. It is important to note, however, that these magnitude measures are obtained from population recording, and do not necessarily reflect the neural activity of the underlying generators.

Across-subject correlation between γ-ERS magnitude and pain-related behavior

To display the between-subject variability of pain-related behaviors elicited by laser stimulation at different stimulus energies (from E1 to E5; Mean ± SD), we averaged scores of pain-related behavior across trials for each subject and each stimulus energy. As displayed in Supplementary Fig. 1, the between-subject variability of pain-related behavior was maximal at energy E2 (left forepaw: 0.2~3.0; right forepaw: 0.3~2.8; left hindpaw: 0.2~3.2; right hindpaw: 0.3~2.9), and relatively large at energy E3 (left forepaw: 2.5~3.3; right forepaw: 2.1~3.4; left hindpaw: 1.8~3.6; right hindpaw: 1.9~3.5). In contrast, due to floor (i.e., E1) and ceiling effects (i.e., E4 and E5), the between-subject variability of pain-related behavior was not well presented in these data (Supplementary Fig. 1).

Considering that across-subject correlation results would be more robust if more stimulus energies were taken into consideration, we analyzed the across-subject correlations between single-subject

averaged magnitude of the TF feature and single-subject averaged scores of pain-related behaviors elicited by nociceptive stimuli at energies E2 and E3. Data at stimulus energies E1, E4, and E5 were not included in the analysis because of their minimal variability caused by floor and ceiling effects. The correlations between estimated time-frequency magnitudes and behavioral scores were expressed as Pearson's r values. As displayed in Supplementary Fig. 6, γ-ERS magnitudes were significantly correlated with pain-related behavior across subjects, regardless of stimulation site (left forepaw: $r=0.61$, $p=0.03$; right forepaw: $r=0.63$, $p=0.03$; left hindpaw: $r=0.68$, $p=0.02$; right hindpaw: $r=0.68$, $p=0.02$).

Supplementary Figures

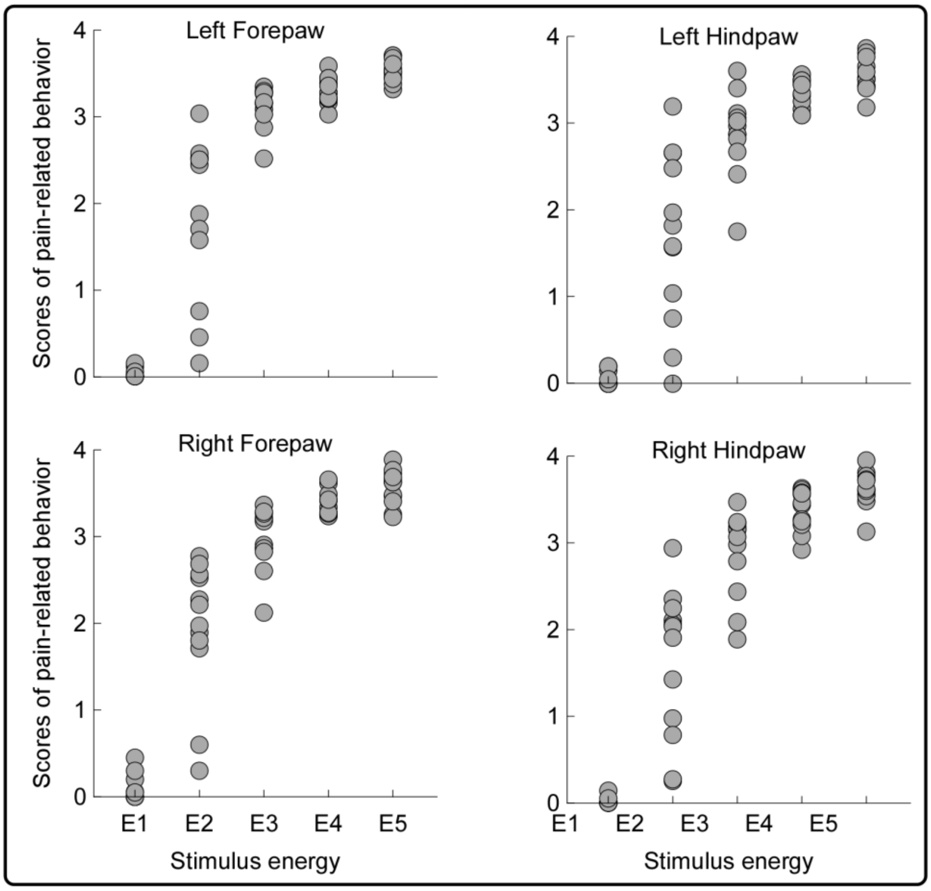

Supplementary Figure 1. Between-subject variability of scores of pain-related behavior. Grey dots represent scores of pain-related behaviors for different subjects, which were elicited by laser stimulation at different stimulus energies (from E1 to E5) and stimulation sites (left forepaw, right forepaw, left hindpaw, right hindpaw). There was clear between-subject variability only in the behavioral responses elicited by E2 and E3, while the other energies showed a floor (E1) or a ceiling (E4 and E5) effect.

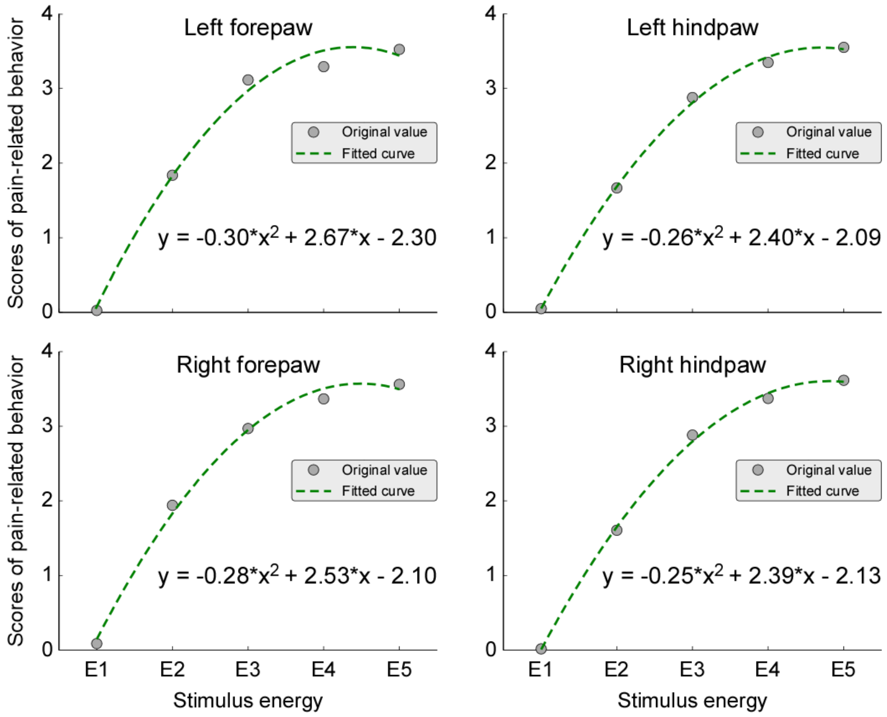

Supplementary Figure 2. The relationship between stimulus energy and pain-related behavior as modeled by a quadratic polynomial function. Grey dots represent average scores of pain-related behaviors elicited by laser stimulation at different stimulus energies (from E1 to E5) and stimulation sites (left forepaw, right forepaw, left hindpaw, right hindpaw). The dashed green lines represent the best fitting curve using the quadratic polynomial function.

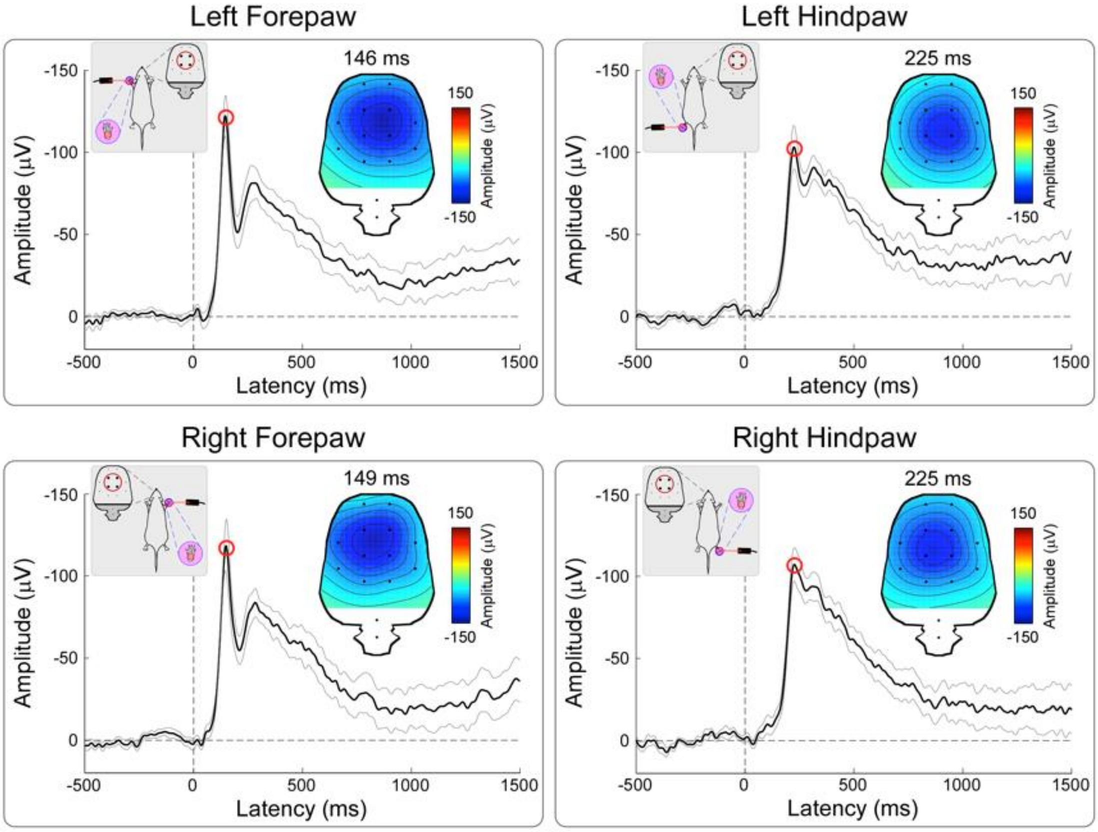

Supplementary Figure 3. Group-level laser-evoked potential (LEP) waveforms and topographies. Displayed signals were measured from four central electrodes (FL2, FR2, PL1, and PR1, circled in red in the insets). Black waveforms represent group-level average LEP responses, and grey waveforms are the SEM across subjects. The scalp topography of the main 'N2' wave, displayed at the peak latency (red circle), is centrally distributed, with a maximum slightly but clearly contralateral to the stimulated territory.

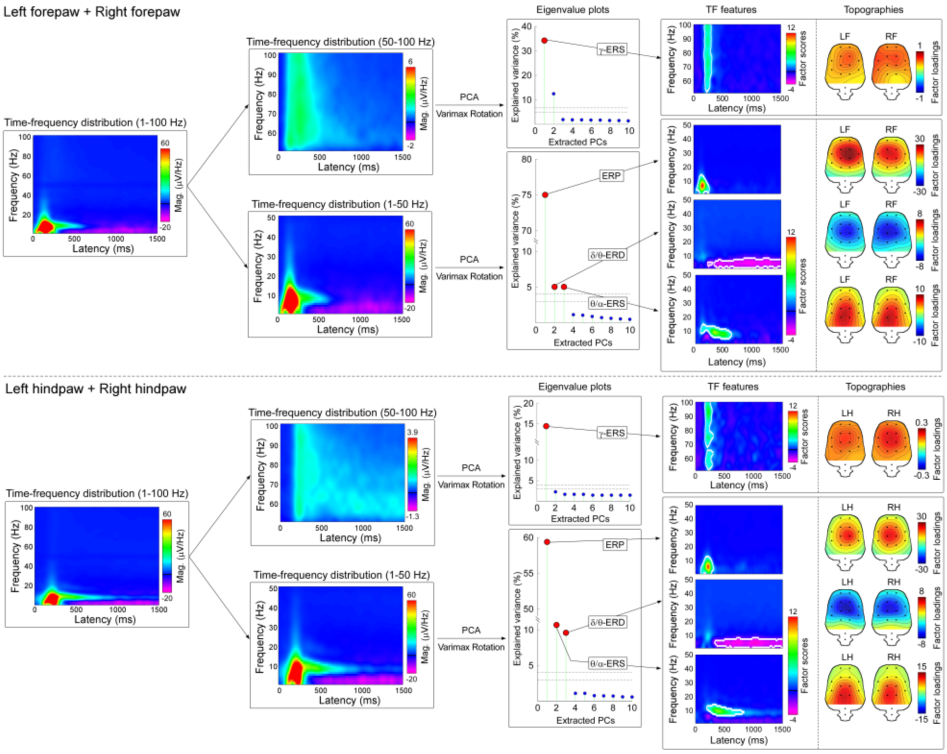

Supplementary Figure 4. PCA with varimax rotation to isolate time-frequency EEG responses, in which EEG trials at stimulus energies E1 were removed. For low frequencies, the first three PCs corresponded to the ERP (located at 47-267 ms and 1-15 Hz for forepaw stimulation, at 83-317 ms and 1-15 Hz for hindpaw stimulation), the δ/θ-ERD (241-1500 ms and 1-9 Hz for forepaw stimulation, 329-1500 ms and 1-9 Hz for hindpaw stimulation), and the θ/α-ERS (83-629 ms and 4-15 Hz for forepaw stimulation, 193-829 ms and 7-15 Hz for hindpaw stimulation). They explained the largest amount of variance of single-trial TFDs (forepaw stimulation: 75.1%, 5.2%, and 5.1% respectively; hindpaw stimulation: 58.9%, 8.6%, and 11.4% respectively). For high frequencies, the first PC corresponded to γ-ERS (175-313 ms and 52-100 Hz for forepaw stimulation, 173-303 ms and 53-100 Hz for hindpaw stimulation) and explained the largest amount of variance of single-trial TFDs (forepaw stimulation: 36.4%; hindpaw stimulation: 13.9%). These results are virtually identical to those reported in the main text including all stimulus energies in the analysis.

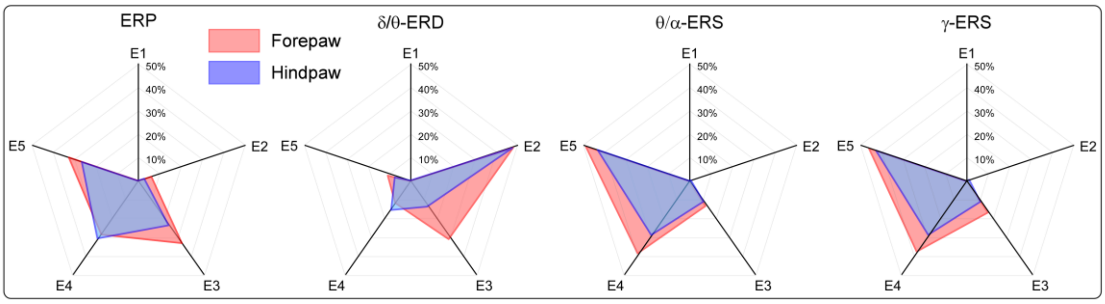

Supplementary Figure 5. Dependency of oscillation magnitude on stimulus energy.

Radar plots show the magnitude of the four PCA-isolated time-frequency features at the five stimulus energies, for forepaw stimulation (light red) and hindpaw stimulation (light blue). The relative strengths of ERP magnitude at stimulus energies E1-E5 were 0.1%, 6.0%, 32.9%, 28.3%, and 32.7% for forepaw stimulation, and 0.1%, 3.7%, 28.2%, 36.5%, and 31.4% for hindpaw stimulation. The relative strengths of δ/θ-ERD magnitude at stimulus energies E1-E5 were 0.6%, 48.7%, 29.8%, 10.7%, and 10.1% for forepaw stimulation, and 1.1%, 57.5%, 15.3%, 17.4%, and 8.7% for hindpaw stimulation. The relative strengths of θ/α-ERS magnitude at stimulus energies E1-E5 were 0.1%, 0.4%, 13.2%, 38.3%, and 48.0% for forepaw stimulation, and 0.01%, 1.0%, 13.1%, 34.4%, and 51.4% for hindpaw stimulation. Similarly, the relative strengths of γ-ERS magnitude at stimulus energies E1-E5 were 0.03%, 0.5%, 16.7%, 37.3%, and 45.5% for forepaw stimulation, and 0.01%, 1.9%, 13.3%, 34.3%, and 50.6% for hindpaw stimulation.

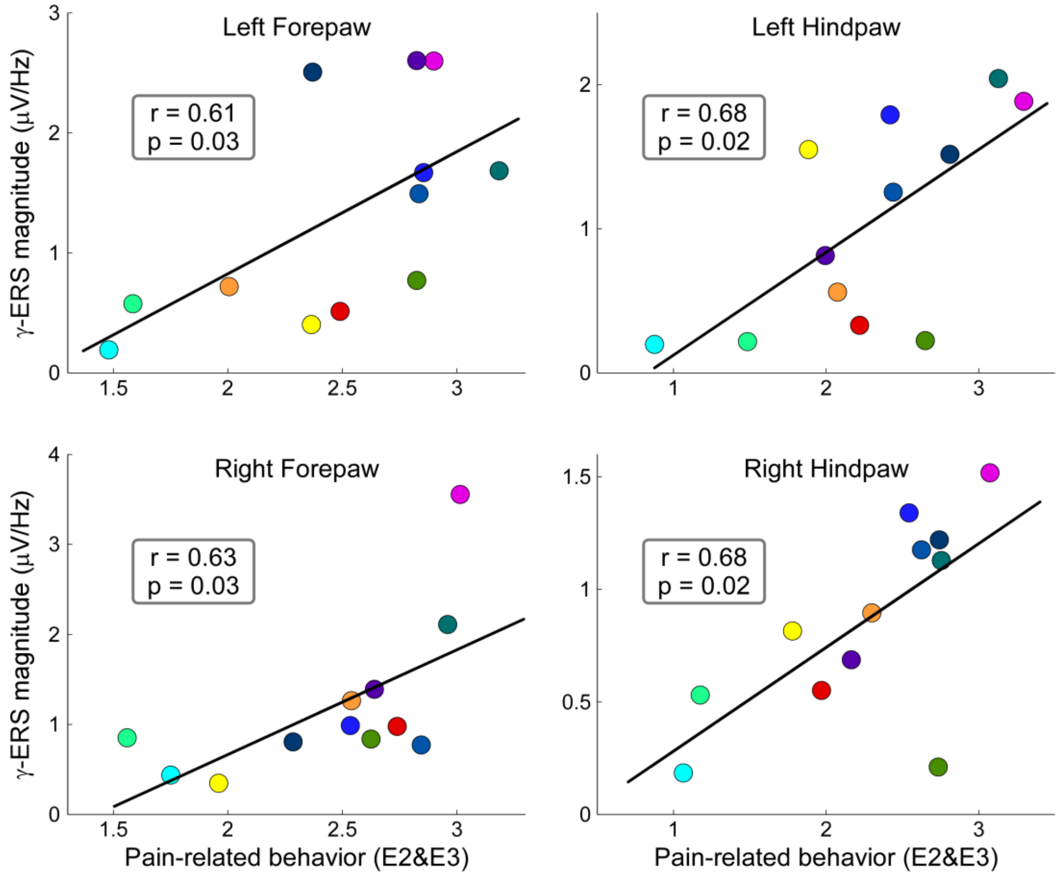

Supplementary Figure 6. Across-subject correlations between γ-ERS magnitude and pain-related behavior, when data of stimulus energies E2 and E3 are pooled. Each dot represents a different subject, and black lines represent the best linear fit. γ-ERS magnitudes were measured at central electrodes contralateral to the stimulated territory, as for the same analysis reported in the main text.

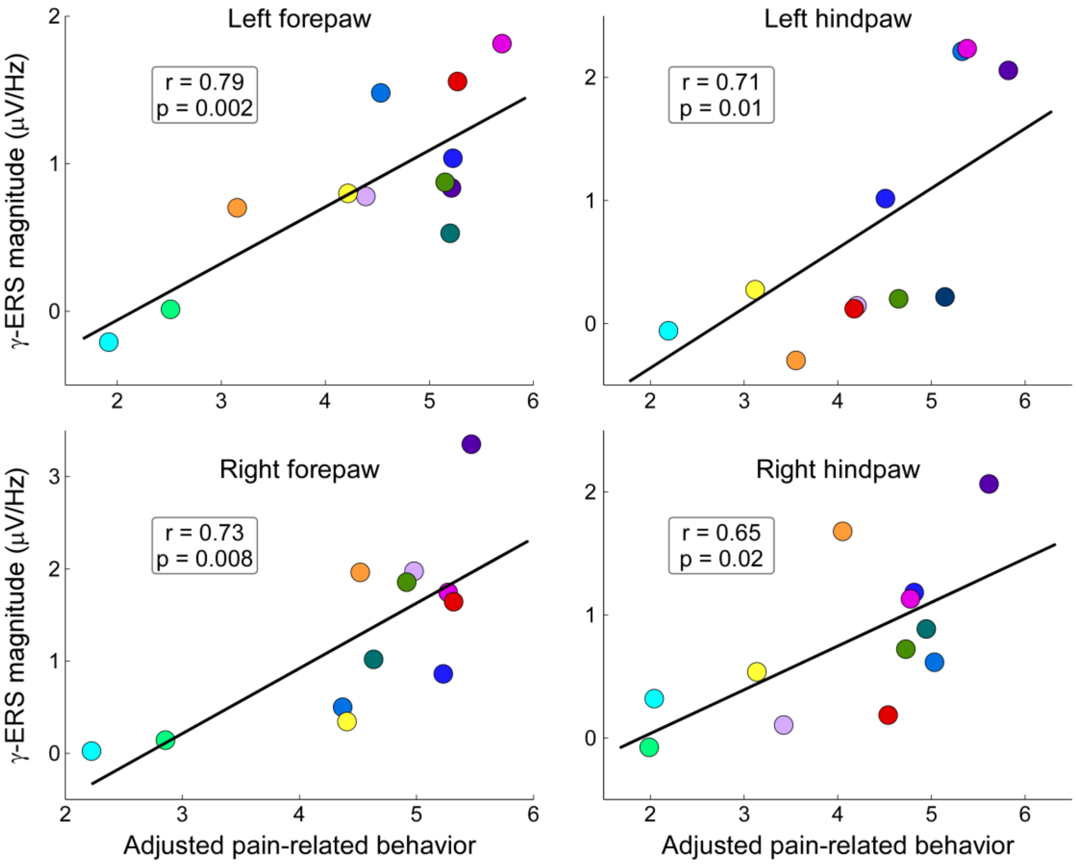

Supplementary Figure 7. Across-subject correlations between γ-ERS magnitude and adjusted scores of pain-related behavior. Scores of pain-related behaviors were adjusted using the modeled quadratic polynomial function. Their relationship with the magnitudes of time-frequency features was assessed using Pearson's R correlation analysis. Each dot represents a different subject, and black lines represent the best linear fit. γ-ERS magnitudes were measured at central electrodes contralateral to the stimulated territory, as for the same analysis reported in the main text.

Supplementary Table

Supplementary Table 1. Correlations between TFD magnitudes and adjusted scores of pain-related behavior, within-subject (trial-by-trial) and between-subjects.

	ERP	δ/θ-ERD	θ/α-ERS	γ-ERS
Within-subject				
LF	**0.45±0.07*****	**-0.14±0.05****	**0.36±0.04*****	**0.53±0.03*****
RF	**0.41±0.06*****	0.02±0.05	**0.33±0.08****	**0.47±0.03*****
LH	**0.42±0.05*****	0.09±0.07	**0.42±0.06*****	**0.40±0.04*****
RH	**0.44±0.06*****	0.08±0.05	**0.41±0.07*****	**0.40±0.05*****
Between-subjects				
LF	**0.59***	-0.43	-0.02	**0.79****
RF	0.57	-0.38	0.40	**0.73***
LH	0.51	-0.30	0.19	**0.71***
RH	0.44	-0.16	0.01	**0.65***

Values express Pearson's r (±SEM); * $p<0.05$; ** $p<0.01$; *** $p<0.001$.

Supplementary references

[1] Paxinos G, Watson C. The rat brain in stereotaxic coordinates: hard cover edition. Academic press. 2006.

[2] Qiao ZM, Wang JY, Han JS, Luo F. Dynamic processing of nociception in cortical network in conscious rats: a laser-evoked field potential study. Cellular and Molecular Neurobiology 2008;28(5):671-687.

[3] Shaw FZ, Chen RF, Yen CT. Dynamic changes of touch- and laser heat-evoked field potentials of primary somatosensory cortex in awake and pentobarbital-anesthetized rats. Brain Research 2001;911(2):105-115.

[4] Xia XL, Peng WW, Iannetti G, Hu L. Laser-evoked cortical responses in freely-moving rats reflect the activity of unmyelinated nociceptive pathways. Neuroimage 2016;128:209-217.
